# Impact of *in Utero* Rat Exposure to 17Alpha-Ethinylestradiol or Genistein on Testicular Development and Germ Cell Gene Expression

**DOI:** 10.3389/ftox.2022.893050

**Published:** 2022-06-02

**Authors:** Laetitia L. Lecante, Bintou Gaye, Geraldine Delbes

**Affiliations:** Institut National de la Recherche Scientifique, Centre Armand-Frappier Santé Biotechnologie, Laval, QC, Canada

**Keywords:** cell sorting, ethinylestradiol (EE2), genistein (GEN), male germ cell, microarray, perinatal, rat, testis

## Abstract

Although the decline in male fertility is believed to partially result from environmental exposures to xenoestrogens during critical developmental windows, the underlying mechanisms are still poorly understood. Experimental *in utero* exposures in rodents have demonstrated the negative impact of xenoestrogens on reproductive development, long-term adult reproductive function and offspring health. In addition, transcriptomic studies have demonstrated immediate effects on gene expression in fetal reproductive tissues, However, the immediate molecular effects on the developing germ cells have been poorly investigated. Here, we took advantage of a transgenic rat expressing the green fluorescent protein specifically in germ cells allowing purification of perinatal GFP-positive germ cells. Timed-pregnant rats were exposed to ethinylestradiol (EE2, 2 μg/kg/d), genistein (GE, 10 mg/kg/d) or vehicle by gavage, from gestational days (GD) 13–19; testes were sampled at GD20 or post-natal (PND) 5 for histological analysis and sorting of GFP-positive cells. While EE2-exposed females gained less weight during treatment compared to controls, neither treatment affected the number of pups per litter, sex ratio, anogenital distance, or body and gonadal weights of the offspring. Although GE significantly decreased circulating testosterone at GD20, no change was observed in either testicular histology or germ cell and sertoli cell densities. Gene expression was assessed in GFP-positive cells using Affymetrix Rat Gene 2.0 ST microarrays. Analysis of differentially expressed genes (DEGs) (*p* < 0.05; fold change 1.5) identified expression changes of 149 and 128 transcripts by EE2 and GE respectively at GD20, and 287 and 207 transcripts at PND5, revealing an increased effect after the end of treatment. Only about 1% of DEGs were common to both stages for each treatment. Functional analysis of coding DEG revealed an overrepresentation of olfactory transduction in all groups. In parallel, many non-coding RNAs were affected by both treatments, the most represented being small nucleolar and small nuclear RNAs. Our data suggest that despite no immediate toxic effects, fetal exposure to xenoestrogens can induce subtle immediate changes in germ cell gene expression. Moreover, the increased number of DEGs between GD20 and PND5 suggests an effect of early exposures with latent impact on later germ cell differentiation.

## Introduction

Evidence is growing that early exposures to endocrine disrupting chemicals (EDCs) can have long term repercussions on reproductive function in mammals, including humans (Mann et al., 2020; Marlatt et al., 2021; Delbes et al., 2022). These chemical contaminants, of both man-made and natural origin, can affect endogenous hormonal pathways by disrupting hormone production or signalling. Epidemiological and experimental studies have associated perinatal exposure to EDCs with a spectrum of male reproductive abnormalities, called the testicular dysgenesis syndrome (TDS) that includes developmental abnormalities such as hypospadias and cryptorchidism as well as testicular cancer and impaired spermatogenesis (Skakkebaek et al., 2016). The molecular mechanisms proposed to cause TDS are primarily alterations of testosterone secretion and functions of somatic cells, inducing indirect effects on differentiating germ cells. However, only a few studies have addressed the hypothesis that germ cells could be a direct target of EDCs. This major gap in knowledge is due to the difficulty of purifying fetal germ cells and the impossibility of discerning direct versus indirect effects of contaminants in *in vivo* studies. Yet these cells express sex steroid receptors early in development (Rouiller-Fabre et al., 2015) and are potential direct targets of EDCs. Moreover, the window of fetal sensitivity corresponds to important germ cell development and events (Manku and Culty, 2015; Rwigemera et al., 2021) associated with major epigenetic and gene expression changes (Law and Oatley, 2020; Rwigemera et al., 2021). It is therefore important to further investigate the effects of EDCs during these windows of sensitivity specifically on germ cells.

In mammals, germ cell differentiation is initiated in the embryo when a small number of cells from the epiblast, the primordial germ cells (PGCs), acquire the germ cell lineage fate and migrate to colonize the genital ridge while actively proliferating (Saitou and Yamaji, 2012). These cells subsequently commit to male or female differentiation pathway depending on the surrounding somatic environment (Wilhelm et al., 2007). In the male gonad, Sertoli cells surround the germ cells, named gonocytes (Clermont and Perey, 1957), forming seminiferous cords. In rats, gonocytes proliferate until gestational day (GD) 17.5 when they enter a quiescent phase in the G0 phase of the cell cycle until post-natal day (PND) 3 when they resume mitosis and differentiate into spermatogonial stem cells (SSCs). The pool of SSCs thus formed will feed spermatogenesis throughout life. These developmental stages occur in parallel with hormonal variations including peaks in androgen and estrogen production at GD18.5, followed by a peak in androgens at PND3 (Habert and Picon, 1984; O'Donnell et al., 2001; Picut et al., 2015). The fetal testis also secrete the anti-mullerian hormone and insulin like factor 3 that are essential for the masculinisation of the reproductive tract (O'Shaughnessy and Fowler, 2011).

Xenoestrogens such as 17alpha-ethinylestradiol (EE2), diethystibestrol (DES), bisphenol A (BPA) or genistein (GE) can be either estrogen receptor agonists or antagonists. These EDCs come from the pharmaceutical industry, manufacturing or natural food respectively. Exposure to DES during pregnancy in humans is a telling example of the adverse effects of *in utero* exposure to xenoestrogen on the reproductive health of the offspring (e.g. increased incidence of cryptorchidism, underdeveloped testes as well as testicular cancer and low sperm counts and quality) (Reed and Fenton, 2013). In rodents, *in utero* exposure to xenoestrogens has been shown to induce developmental abnormalities in the male reproductive system, affecting steroidogenesis, somatic differentiation and gonocyte proliferation and/or differentiation resulting in a predisposition to infertility in adulthood (Reviewed in Lombo and Herraez, 2021; Walker et al., 2021; Delbes et al., 2022). In this respect, a window of sensitivity of the fetal testis to estrogen receptors agonists has been identified (Delbes et al., 2007). The hypothesis of a direct estrogenic effect on the developing reproductive system is justified by the presence of the two distinct intracellular receptors: estrogen receptor alpha (ERα) and ERẞ in the developing testis (Rouiller-Fabre et al., 2015; O'Donnell et al., 2001) and alteration of fetal testis development in knockout mice for ERα (Delbes et al., 2006) and ERẞ (Delbes et al., 2005). To explain the immediate effects resulting in latent reproductive effects, Naciff et al. (Naciff et al., 2005) tested the effect of exposure to a dose-range of EE2, GE or BPA, in Sprague Dawley rats, on testicular gene expression. Although they did not observe any morphological effect, they identified changes in gene expression after exposure to medium to high doses, but little commonalities between treatment groups. Major affected pathways were related to steroidogenesis but many uncharacterised transcripts were also identified. Overall, this study provided important information on the testicular transcripts potentially affected by xenoestrogens and the dose-dependent nature of these effects. However, due to overrepresentation of somatic RNAs it did not reveal any germ cell-specific effects.

Here, we were inspired by the methods used in Naciff et al. (Naciff et al., 2005), to further test the immediate effects of *in utero* exposure to estrogen receptor agonists on germ cell gene expression. To that end, we took advantage of a transgenic rat expressing the green fluorescent protein (GFP) specifically in germ cells (Cronkhite et al., 2005) allowing efficient purification of rat perinatal germ cells (Rwigemera et al., 2017; Rwigemera et al., 2021). EE2 and GE were selected as xenoestrogens with different potency and affinity for the two ERs (Kuiper et al., 1997). We have characterized the cellular and molecular effects of exposure from GD13 to GD19, reporting alteration in germ cell gene expression related to olfactory transduction and non-coding RNAs despite no histological changes.

## Materials and Methods

### Chemicals

Corn oil (#C8267) and 17α-ethinylestradiol (#E4876; CAS 57-63-6) were purchased from Sigma–Aldrich Canada Ltd. (Oakville, Ontario, Canada). Genistein (#G-6055; CAS 446-72-0) was purchased from LC- laboratories (Woburn, MA, United States). All chemicals were certified with a purity ≥98% by the provider. Concentrated stock solutions of EE2 (160 μg/ml) and GE (800 mg/ml) were prepared in dimethyl sulfoxide (DMSO), aliquoted and stored in amber glass bottles at -80°C. Working solutions were prepared daily by diluting stock solutions 200 times in corn oil (0.5% final DMSO concentration).

### Animals

Transgenic Sprague-Dawley rats expressing germ cell-specific GFP (GCS-EGFP) (Cronkhite et al., 2005) were used as previously described (Rwigemera et al., 2021). Rats were housed on a 12L:12D cycle and were fed with commercial food (Teklad global 18% protein, Envigo, Madison, WI) and tap water *ad libitum*. All animal studies were conducted in accordance with the guidelines set out by the Canadian Council of Animal Care (CCAC) and approved by the Institutional Animal Care and Use Committee at the INRS (Protocol #1405-10 and #1902-01). Fifteen weeks-old virgin females were caged with one 15-25 weeks-old male overnight. Vaginal smears were done the following day to identify sperm-positive females. From that day defined as GD0, sperm-positive females were housed individually. On GD13, pregnant dams were randomly assigned to treatment groups (n = 5 per group). Until GD19, each dam was weighed daily and gavaged with 2 μg/kg/day of EE2 or 10 mg/kg/day of GE in corn oil; equivalent vehicle concentration (0.5% DMSO) in corn oil was administered to control animals. On GD20, five randomly designated pregnant dams per group were euthanized by CO_2_ asphyxiation followed by cervical dislocation and fetuses were immediately removed from the uterus. Other litters were kept after birth for sampling at PND5. Any developmental abnormality following global anatomical observations of the offspring as well as stillbirth were recorded. Number of pups per litter as well as sex ratio, placenta and body weights were determined. For each pup, the anogenital distance (AGD) was measured from the centre of the anus to the tip of the penis or to the vaginal opening, using a calliper (Fisher Scientific) (Table 1). To ensure reproducibility, the same investigator did all measurements, blind to the treatment group.

**TABLE 1:** Effect of *in utero* exposure to ethynil estradiol (EE2) or gensitein (GE) on perinatal development. Females were exposed by gavage from gestational day (GD) 13 to 19. Progeny was sacrificed on GD20 (n = 5 litters) or post-natal day 5 (PND5, n = 4 litters). Male and females were counted and weighted. Ano-genital distance was measured from anus to tip of genital tubercle and gonads were sampled and weighted. *p < 0.05 compared to control using a non-paired *t*-test.

		Ctrl	EE2	GE
	Maternal weight gain during treatment (g)	47.67 ± 1.58	41.00 ± 1.38*	43.89 ± 1.2
GD20	Number of live fetuses per litter	12.20 ± 0.86	12.00 ± 0.55	12.60 ± 0.51
	Male fetuses weight (g)	3.54 ± 0.10	3.52 ± 0.08	3.52 ± 0.06
	Female fetuses weight (g)	3.42 ± 0.07	3.44 ± 0.08	3.37 ± 0.07
	Anogenital distance males (mm)	4.13 ± 0.13	3.98 ± 0.14	3.91 ± 0.09
	Anogenital distance females (mm)	2.37 ± 0.12	2.27 ± 0.15	2.28 ± 0.07
	Testis weight (mg)	1.43 ± 0.05	1.48 ± 0.06	1.39 ± 0.05
	Ovary weight (mg)	0.21 ± 0.01	0.24 ± 0.05	0.21 ± 0.01
PND5	Number of live pups per litter	11.75 ± 0.63	10.75 ± 1.25	10.75 ± 0.48
	Male pups weight (g)	10.28 ± 0.32	10.15 ± 0.48	9.97 ± 0.39
	Female pups weight (g)	9.84 ± 0.29	9.72 ± 0.44	9.55 ± 0.37
	Anogenital distance males (mm)	6.64 ± 0.24	6.75 ± 0.33	6.51 ± 0.27
	Anogenital distance females (mm)	3.66 ± 0.13	3.57 ± 0.24	3.41 ± 0.16
	Testis weight (mg)	6.87 ± 0.24	7.03 ± 0.34	6.71 ± 0.12
	Ovary weight (mg)	0.48 ± 0.04	0.37 ± 0.04	0.36 ± 0.06

### Blood and Tissue Collection

Fetuses and pups were decapitated. Blood was collected from the neck and pooled per sex per litter. Serum collected was kept at -80°C. Fetuses and newborn rats were placed on ice prior to dissection under a binocular microscope. Gonads were collected and pooled per sex and weighed on a precision analytical scale. Measurement obtained was divided per number of gonads in order to obtain a mean testis or ovary weight per litter. Two testes per litter were fixed for further histological analyses, and the remaining testes from the same litter were pooled and used to purify germ cells by flow cytometry cell sorting (FACS).

### Serum Testosterone Levels Measurement

Testosterone concentrations were determined from pooled male serum using the testosterone ELISA kit (Immuno-Biological Laboratories (IBL)—Minneapolis, MN, United States, #IB79106) according to the manufacturer’s instructions. Given the very small volume available, only one measurement could be done for GD20 samples while PND5 samples were run in duplicates. No sample dilution was done.

### Immunohistochemistry

Testes were fixed for 24 h in Bouin’s fluid, dehydrated with ethanol, and embedded in paraffin. Each 10th 5 µm-thick sections of GD20 testes and two representative sections of PND5 ones were mounted on slides. Gonocytes were immunostained with a mouse anti-heat shock protein 90 (HSP90) antibody (1:100; BD Bioscience #610419) and Leydig cells with a chicken anti-3ẞ -hydroxysteroid dehydrogenase (3β -HSD) antibody (1:4000; Immune biosolution #Y000098-002). In short, after dewaxing and rehydration, slides were incubated in a 3% H2O2 aqueous solution for 10 min at room temperature (RT) (21–25°C) in order to inhibit endogenous peroxidase activity. A heat-induced antigen retrieval step was performed by submerging slides in a pH 9.0 Tris 10 mM EDTA 1 mM Tween 20 (0.05%) buffer and microwaved until the solution came to a boil and then for 7 min at 30% power. Slides were left to cool down for 15 min and rinsed in doubled-distilled water. From that moment onward, incubations were performed in a humidified chamber and sections were washed with phosphate buffered saline (PBS) solution between each step. Sections were incubated with a blocking solution containing 5% Bovine Serum Albumin (Sigma-Aldrich, #A4503, Oakville, Ontario, Canada) in PBS, at RT, for 1 h, to avoid non-specific antibody binding. Thereafter, sections were incubated overnight at +4°C with the primary antibody diluted in blocking solution. Primary antibody was detected by incubation at RT with biotinylated anti-mouse (1:200; Vector PK4002) and the avidin–biotin–peroxidase complex (Vectastain Elite ABC kit; Vector Laboratories, Burlingame, CA) or horseradish peroxidase (HRP) coupled anti-chicken (1:200; Immune biosolution #Y01038-HRP) secondary antibody diluted in blocking solution. Thus, immunolabeling was developed with 3,3′-diaminobenzidine tetrahydrochloride (DAB Substrate Kit, SK4100, Vector Laboratories) preceded with a streptavidin-HRP (Vectastain ABC kit, Vector Laboratories, Burlingame, CA, United States) incubation when necessary. Tissues were counterstained with hematoxylin (Surgipath #01521), dehydrated and coversliped using Permount medium.

### Stereology

Stained tissue sections were observed and captured at ×40 magnification under a light microscope (Zeiss, Primo Star) equipped with a computational-assisted camera (Axiocam ERc5s, Zeiss). Outlines of 50-100 testis cords representing a minimum of 50,000 µm2 distributed on 2-5 randomly chosen sections per sample were delimited with ZEN imaging software (Carl Zeiss, blue version). The number of gonocytes (HSP90-positive cells) and Sertoli cells (HSP90-negative cells) were assessed within the same seminiferous cords to determine each cell type density (cell number/µm^2^of testis cord). Counting was cell nucleus based and blind to the treatment group.

### Germ Cell Purification

Remaining testes from the same litter were decapsulated and single cell suspensions were obtained following a 2-step enzymatic digestion procedure as previously described (Rwigemera et al., 2017). A trypan blue-stained subset of this single cell suspension was used to determine cell concentration and viability. Cells were then diluted to reach an optimal concentration of 5-10 million cells/ml for cell sorting using a FACSJazz flow cytometer (BD Biosciences, San Jose, CA) at an event rate of ∼2,500 events/sec as described in (Rwigemera et al., 2017). After sorting, the GFP-positive cell fraction was washed with Hanks’ Buffered Salt Solution and counted. The total number of cells collected, viability and purity per fraction (percentage of GFP-positive cells) were calculated using a hemocytometer under a TiS fluorescent microscope (Nikon, Mississauga, Ontario, Canada). Sorted cells were finally aliquoted, pelleted, snap frozen in liquid nitrogen and stored at −80°C until RNA extraction.

### RNA Extraction and Gene Expression Microarray

Cell pellets of 50,000 to 100,000 cells were thawed on ice for 5–10 min prior to extracting total RNA using the Arcturus PicoPure RNA isolation kit (ThermoFisher Scientific, Saint-Laurent, Quebec, Canada, #KIT204) as previously described (Rwigemera et al., 2021). Following RNA quantification with the NanoDrop One (Thermo Scientific, Wilmington, DE, United States), the RNA integrity number (RIN) was determined using a bioanalyzer. Only samples with RIN above eight were used for gene expression analysis (n = 3/group from different litters) based on GeneChip Rat Gene 2.0 ST array (ThermoFisher Scientific, Saint-Laurent, Quebec Canada) in collaboration with the Genome Quebec Innovation Centre, according to the manufacturer’s recommendations. All .cel files generated were used for data normalization and analysis using the Transcriptomic Analysis Console (TAC) software provided by ThermoFisher Scientific (release 4.0.2). Gene expression data used in this manuscript have been deposited in the ArrayExpress database at EMBL-EBI (www.ebi.ac.uk/arrayexpress) under accession number E-MTAB-11591. Differentially expressed genes (DEGs) were selected using different set of parameters: 1) fold change (FC) = 1.5 and a false discovery rate adjusted *p*-value (FDR) < 0.05; 2) FC = 2 and *p*-value<0.05; 3) FC = 1.5 and *p*-value<0.05 (Table 2). Detailed gene clustering and DEGs enrichment according to KEGG pathway analysis was further done using their Entrez Gene ID in the David Bioinformatic resources (Huang da et al., 2009).

**TABLE 2:** Number of differentially expressed genes (DEGs) using different sets of statistical parameters.

Age	GD20		PND5	
Treatment	EE2	GE	EE2	GE
FDR<0.05; 1.5-fold change	0	0	0	0
*p*-value<0.05; 2-fold change	16	11	28	23
*p*-value<0.05; 1.5-fold change	149	128	285	207

### Statistical Analysis

For all analyses, the litter was considered as one unit, therefore n represents the number of litters considered for the parameter assayed. All values are means ± SEM of n = 3-5. GraphPad Prism 6 (GraphPad Software, La Jolla, CA, United States) was used to analyse all phenotypic parameters and immunohistochemistry quantification data that were analysed using a Kruskal–Wallis test followed by a Mann-Whitney test. For transcriptomic data, statistical analyses were done using an eBayes ANOVA Method on the TAC software.

## Results

### Maternal and Fetal Toxicity

We first investigated the effects of *in utero* exposures to EE2 or GE on pregnancy and developmental endpoints including maternal weight gain, number of live pups, sex ratio, male and female fetuses’ weights (Table 1). EE2-exposed dams gained significantly less weight compared to controls, yet the number of total and live offspring per litter, counted both at GD20 and PND5, were not affected by gestational exposure to EE2 or GE. Furthermore, none of the treatments impacted offspring weight and AGD with respect to either perinatal age or sex (Table 1). Measurement of gonadal weights, reported per testis or per ovary from a pool per litter, also showed no significant change after treatments (Table 1).

### Effects on Steroidogenesis and Testis Development

**FIGURE 1:**
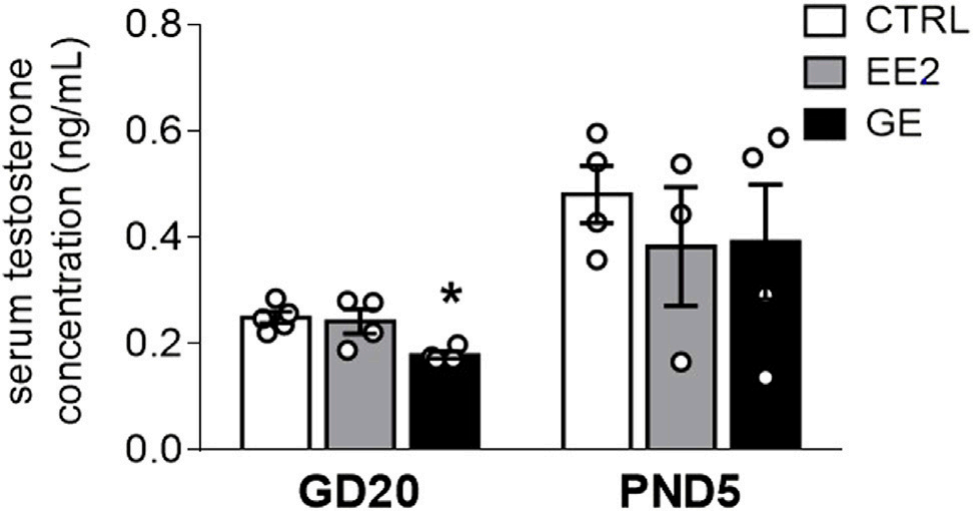
Effects of *in utero* exposure to EE2 or GE on steroidogenesis. Mean testosterone concentration measured within pooled serum of males sampled on GD20 or PND5 following *in utero* exposure to either vehicle (CTRL), ethinylestradiol (EE2) or genistein (GE) are represented as the mean ± SEM. Individual values are also displayed on the graph (n = 3-4 from different litters/group). * indicates a *p*-value<0.05 when compared to controls.

We next assayed whether maternal exposures to EE2 or GE would interfere with testicular hormone secretion. GE significantly decreased serum testosterone levels by 28% compared to control at GD20 (Figure 1). However, this alteration was no longer observed at PND5. EE2 did not affect this parameter compared to the control group whatever the time point (Figure 1).

**FIGURE 2:**
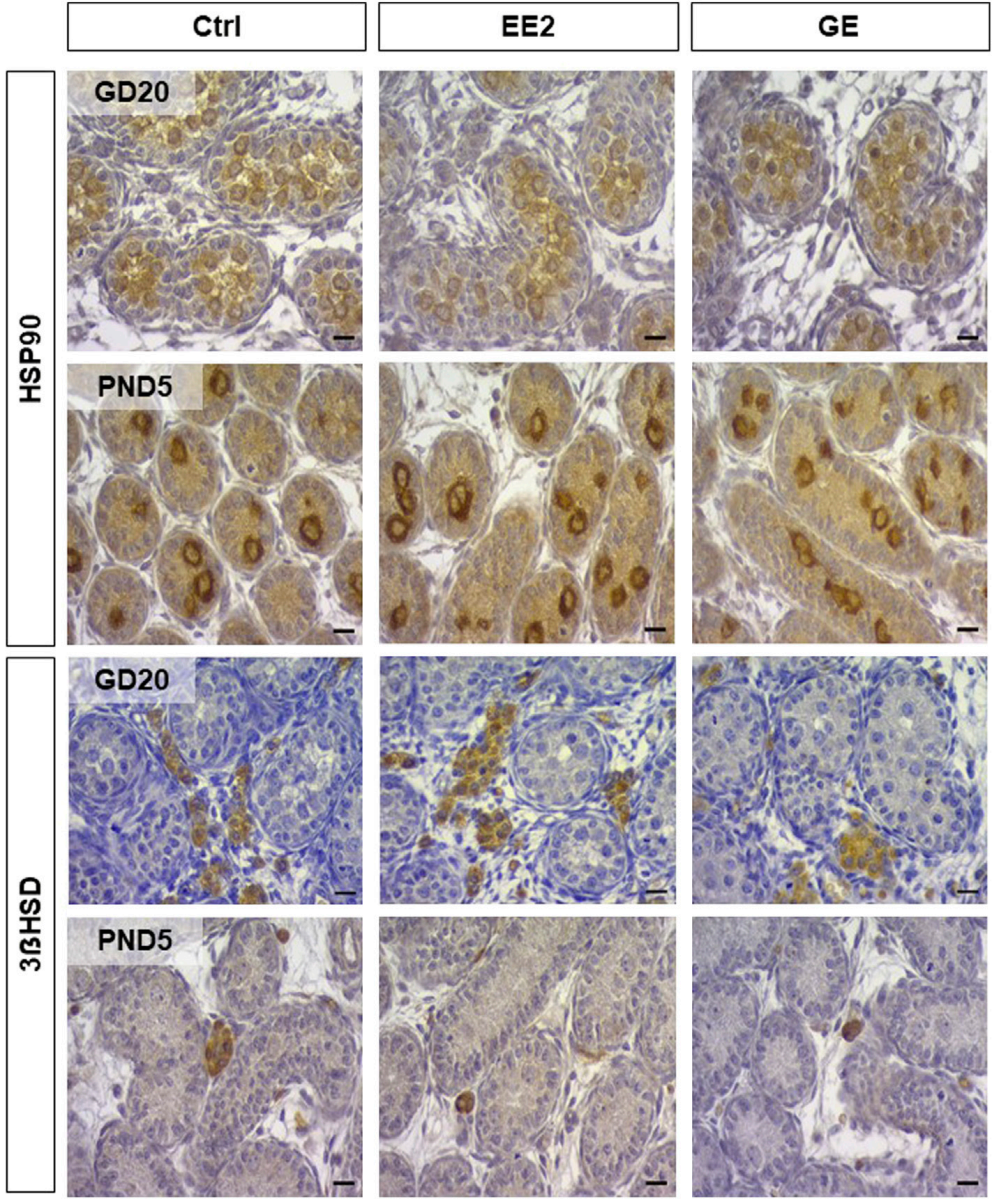
Analysis of testicular histology after *in utero* exposure to EE2 or GE. Representative illustrations of testicular sections collected after exposure to ethynilestradiol (EE2) or Genistein (GEN) at Gestational Day 20 (GD20) and post-natal day 5 (PND5). Top rows illustrate staining for Heat shock protein 90 (HSP90) in developing germ cells, while bottom rows illustrate staining for 3ẞ Hydroxy-steroid deshydrogenase (3ẞHSD) in Leydig cells. Scale bar represents 10 µm.

**FIGURE 3:**
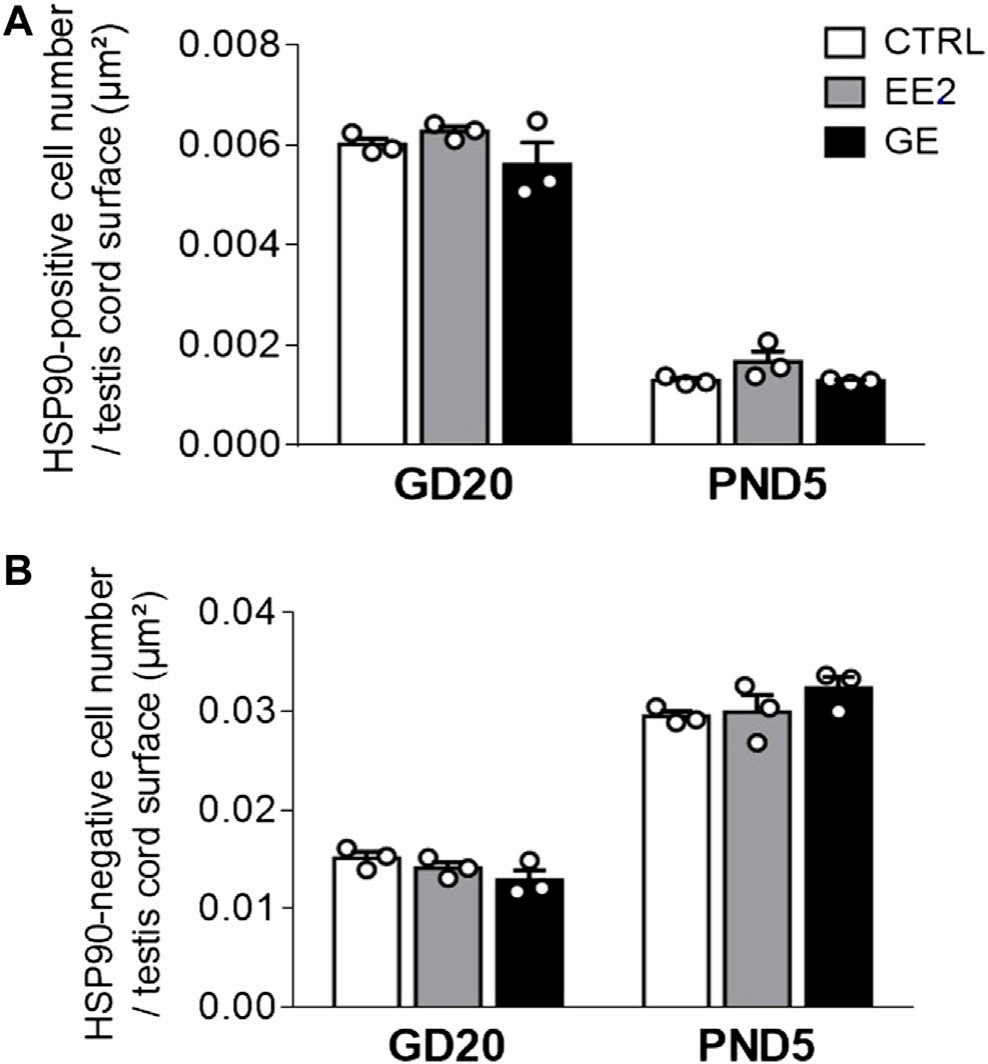
Effects of *in utero* exposure to EE2 or GE on testis development. Mean density of **(A)** HSP90-positive cell number and **(B)** HSP90-negative cell number per testis cord surface within testes sampled on GD20 or PND5 following *in utero* exposure to either vehicle (CTRL), ethinylestradiol (EE2) or genistein (GE) are represented as mean ± SEM. Individual values are displayed on the graph (n = 3 from different litters/group).

Provided that a fine balance between germ and supporting-Sertoli cells is crucial to ensure optimal gametogenesis, we assessed whether gestational exposures to EE2 and GE could affect testis cords organization and cellular density. The global testicular architecture was not affected by any exposure at both sampling ages (Figure 2). Germ cells HSP90 immunolabellings were comparable between exposed groups and age corresponding controls (Figure 2). Similarly, there was no indication of major effect after exposure to EE2 or GE based on observation of fetal Leydig cells after immunolabelling for 3βHSD (Figure 2). Further quantification of HSP90-positive (Figure 3A) and HSP90-negative cells per seminiferous cord surface (Figure 3B) showed no significant effect of both gestational exposure on germ cells and Sertoli cells densities at both perinatal stages assessed.

### Effects on Germ Cell Gene Expression

**FIGURE 4:**
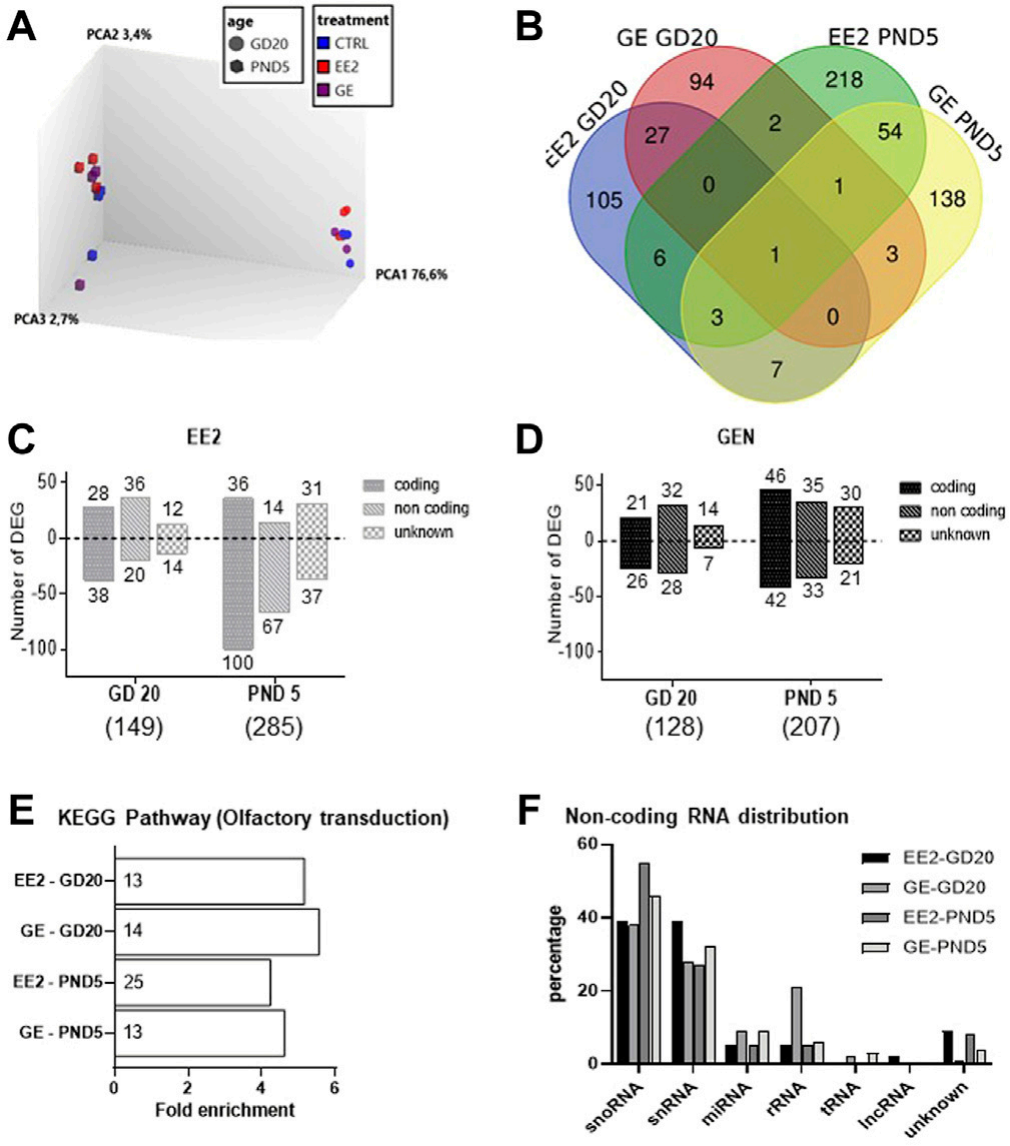
Analysis of microarray data for germ cells gene expression after *in utero* exposure to EE2 or GE. **(A)** 3D PCA plot of all samples representing the global differential gene expression patterns in GFP-positive sorted cells after treatment at the two sampling ages **(B)** Total numbers of differentially expressed genes (DEGs) and overlaps after exposure to EE2 or GE at the two sampling stages. **(C)** Numbers of up- and down-regulated DEGs after exposure to EE2 at the two sampling ages **(D)** Numbers of up- and down-regulated DEGs after exposure to GE at the two sampling ages. **(E)** Enrichment analysis revealing common enrichment for the KEGG pathway rno04740- Olfactory transduction (the number of DEGs is specified for each treatment group and stage, in each histogram bar) **(F)** Distribution of DEGs according to non-coding RNA types, in the different treatment and age groups.

To address whether gestational exposure to EE2 or GE during the masculinization window affects gene expression specifically in germ cells, we purified GFP-positive cells from pooled testes per litter. We ensured that cell viability was above 85% before and after cell dissociation (Supplementary Table S1). Moreover, the average purity, evaluated by the percentage of GFP-positive cells in the sorted fractions, was always above 85% (Supplementary Table S1). Consistently with the fact that the density of HSP90-positive cells was not affected by the treatments, the average number of GFP-positive cells collected reported per testis was not affected by the treatments at both sampling ages (data not shown). Transcriptomic profiling was then done on RNA extracted from GFP-positive cells, using the Rat Gene 2.0 ST array on which 610,400 probe sets are present for an estimated 28,407 transcripts covered (median of 22 probes/transcript). A principal component analysis of global gene expression showed that age was the most differentiating parameter (Figure 4A).

A detailed analysis of DEGs for each treatment at each age, revealed 149 and 128 genes for which expression significantly varied after EE2 or GE treatment, respectively at GD20 while 287 and 207 were affected at PND5. These DEGs were identified using cut-off values of 1.5-fold change and *p*-value<0.05 while more stringent cut-off values generated very few to no DEG (Table 2). A Venn diagram of the four groups showed very limited overlap of DEGs (Figure 4B). Indeed, only 5.4% and 7.9% of DEGs were common between treatments at GD20 and PND5, respectively. Also, not even 1% of DEGs were common to both ages within the same treatment group. The only transcript common to all groups was coding for sperm motility kinase 2A (Smok2a).

The DEGs distributions are shown in Figures 4C,D, respectively, specifically at GD20 or PND5 for each treatment. Lists of DEGs are also detailed in Supplementary Tables S2–5. While treatment ceased the day before sampling at GD20, it is interesting to note that the total number of DEGs increased in both treatment groups from GD20 to PND5. To deepen our analysis, we were able to distinguish 3 categories of DEGs for each list, the majority being protein-coding and non-coding (nc) RNAs followed by unknown transcripts, each of which increasing from GD20 to PND5 (Figures 4B,C).

For protein-coding genes, we then tested for any significant enrichment according to KEGG pathways. We could only include 65–80% of the coding transcripts for which there is a Gene ID recognized by DAVID in this analysis. All four groups were significantly enriched for the rno04740 pathway corresponding to olfactory transduction. (Figure 4E). This enrichment is exclusively due to the presence of transcripts encoding olfactory receptors (Olr) in the four lists. Their numbers in each group are presented in the histogram bars in Figure 4E. Analysis of common Olr between the four groups showed little overlap (Supplementary Figure S1A) but identified 3 Olr at GD20 (Olr1355, Olr1504, Olr1632) and 3 others at PND5 (Olr1335, Olr1520, Olr295) common in both treatments. Olr792 was affected at both stages but only by EE2.

In parallel, analysis of ncRNA according to RNA type revealed similar distribution among the groups, with highest proportions of small nucleolar (sno) and small nuclear (sn) RNA (Figure 4F). Analysis of commonalities between the four groups showed some degree of overlap, especially per stage of sampling (Supplementary Figure S1B).

## Discussion

This study was designed to characterise the impact of estrogen receptor agonists on testicular development and more specifically on gene expression in perinatal germ cells. The GCS-EGFP rat strain used allowed rapid purification of germ cells by FACS and facilitated comparison with the literature, as the Sprague Dawley rats have been most commonly used in reproductive and developmental toxicity studies addressing the impact of xenoestrogens (Naciff et al., 2005). Timed-pregnant females were exposed from GD13 to GD19, a time of exposure that included the fetal proliferative period, when testes are highly sensitive to variation of estrogens (Delbes et al., 2007) and androgen signalling (Welsh et al., 2014). The choice of exposure by gavage was intended to mimic as closely as possible the main route of exposure to EDCs in humans. Finally, doses were selected on the basis of previous studies showing gene expression changes in fetal testis (Naciff et al., 2005) and no major developmental toxicity (Delclos et al., 2009) after *in utero* exposure to increasing doses of EE2, or GE. In our study, the human equivalent doses correspond to 1.6 mg/kg/day and 0.32 μg/kg/day for GE and EE2 respectively (human equivalent dose (in mg/kg) = rat dose (in mg/kg) × (rat Km/human Km) with human Km 37) and rat Km 6) (Reagan-Shaw et al., 2008)), which represent 10-100 times the regular sources of genistein from the western diet and an equivalent of the EE2 intake from the contraceptive pill (Mulligan et al., 2007; van den Heuvel et al., 2005). In the present study, other sources of xenoestrogens were reduced by using a diet formulated with reduced soy content and consequently minimized levels of isoflavones (the main phytoestrogens present in the rodent diet). However, we were unable to control intakes of plasticizers (cage and bottle) that might have contributed to a basal level of endocrine disruption and diminished the differential observed effects. Under these conditions, our data showed that these treatments did not induce any obvious developmental toxicity; no effect was observed on litter size, sex ratio or offspring body weights. Yet, consistently with other studies showing that estrogens are anorectics and reduced food intake, we observed a relatively modest but significant decrease in weight gain in EE2-exposed females (Wallen et al., 2001; Delclos et al., 2009). This effect was not observed in GE-exposed females which is in accordance with comparative studies showing higher magnitude of the effect in the EE2-exposed animals compared to GE (Wallen et al., 2001; Delclos et al., 2009). This difference is probably due to the lower affinity of genistein for ERs than EE2 (Kuiper et al., 1997).

Offspring sampling was done on GD20, at the end of treatment, and on PND5, when the SSCs pool arises (Manku and Culty, 2015; Law and Oatley, 2020). Analysis of the AGD, gonadal weights and the circulating testosterone levels did not reveal major changes. The minor yet significant decrease in testosterone levels in GE-exposed male fetuses was not associated with decreased AGD in males. Such observation is in line with the fact that only marked drops in testosterone levels will induce shorter male AGD (Schwartz et al., 2019). The difference in effect on circulating testosterone between EE2 and GE at GD20 could be due to different fetal pharmacokinetics between the two compounds. To our knowledge, their distribution in the fetal gonads following maternal exposure is not yet well characterized. Furthermore, histological analysis of the testes revealed no major structural effect, as previously described (Naciff et al., 2005). The quantitative analysis presented here nicely illustrates the expected decrease in germ cell density associated with the increase in Sertoli cell density between the two sampling stages. Importantly, neither treatment had any effect on the composition of these 2 cell types in seminiferous cords. Furthermore, observations of interstitial clusters of fetal Leydig cells did not reveal any major changes between the two treatments and controls.

To test the impact of exposures specifically in the developing male germ cells, sorted GFP-positive cells were used. We have previously shown that this strategy allows germ cell sorting with high purity, viability and enrichment in germ cell-specific transcripts (Rwigemera et al., 2021). Gene expression was studied using the Affymetrix Rat Gene 2.0 ST microarray revealing hundreds of DEGs per group under statistical cut-off of *p*-value<0.05 and a 1.5-fold difference. To our knowledge, there are only few developmental exposure studies on the perinatal germ cell transcriptome with which to compare our data, since most of these studies have investigated long-term inter- and transgenerational effects on spermatozoa, which are easier to collect and purify (Walker et al., 2020; Skinner, 2016; Ahmad et al., 2014; Robaire et al., 2022). Yet, our data are consistent with other developmental and transgenerational studies on EDCs (Iqbal et al., 2015; Skinner et al., 2013). For example, one of the parameters studied by Iqbal et al. (Iqbal et al., 2015) was gene expression in FACS-sorted murine germ cells at GD17.5 after gestational exposure to BPA, di(2-ethylhexyl) phtalate (DEHP) or Vinclozolin. Using cut-off values of 1.5-fold change and FDR-p<0.05, they identified seven and two DEGs after DEHP and vinclozolin exposure, respectively, with no DEG detected in BPA-exposed germ cells. However, as in the present study, when the statistical thresholds were relaxed to a *p*-value<0.05 and a 1.5-fold difference, they found an average of 264 DEGs per condition. Similarly, Skinner’s group study (Skinner et al., 2013) of the transgenerational effect of *in utero* exposure to vinclozolin on StaPut-purified rat F3 embryonic germ cells, used *p*-value<0.05 and 1.5-fold difference identifying hundreds of DEGs. Taken together, our data suggest that under our experimental conditions, with these doses, the effects observed in germ cell gene expression are minor.

Analyses of DEGs in the present study revealed that very few were commonly affected by EE2 and GE, suggesting distinct effects of these xenoestrogens. This is consistent with what has been found by others (Naciff et al., 2005; Delclos et al., 2009) and may be due to their different affinities for the two estrogen receptors (Kuiper et al., 1997; Gutendorf and Westendorf, 2001). However, we observed one DEG common to all groups, that was Smok2A, a testis specific gene characterised. Smok2A is part of a protein kinase gene family with reduced kinase activity involved in sperm motility regulation (Herrmann et al., 1999). Yet to our knowledge, its earlier role in differentiating male germ cells is not known. Further studies on how early impairment of Smok2A expression could have long-term impact on sperm motility could identify the mechanism underlying known long-term effect of gestational exposure to estrogen receptor agonist on sperm motility (Ahmad et al., 2014).

In parallel, we have identified common enrichment for the olfactory transduction pathway after both EE2 and GE treatment. Such enrichment had already been identified by the Skinner’s study cited above (Skinner et al., 2013). Since the study from that group focused on vinclozolin, an antiandrogenic compound, in the F3 germline, whereas we studied the F1 germline after exposure to estrogen receptor agonists, this may suggest Olr genes are common targets of EDCs in developing germ cells over generations. Expression of Olr genes in mammalian testis have been described (Kang and Koo, 2012) and studies of their genomic structure revealed non classical promotors with unique epigenetic signature and regulation (Clowney et al., 2011) suggesting a need for further study of their response to contaminants. OLRs are G protein coupled receptors involved in chemotaxis. Because they can be involved in Calcium influx and are present on the sperm head membrane, they have been suggested to play a role in sperm chemotaxis, capacitation and motility (Ali et al., 2021). Yet, to our knowledge, their role in developing germ cells is unknown. Interestingly, the Rat Gene 2.0 ST chip contains 1,194 probes designed against different OLR-encoding genes that are present throughout the genome. It will be interesting to use these data to further investigate expression patterns in male germ cells over time.

We also observed common effects on snoRNAs that are encoded in the introns of genes and described as a large class of non-coding RNAs. Classically snoRNAs guide ribosomal RNA and its processing but they have been suggested to play other key functions such as the regulation of chromatin structure or splicing of pre-messenger RNA (Dupuis-Sandoval et al., 2015). The regulation of their expression is coupled to host genes making it difficult to understand their response to estrogen receptor agonists as highlighted in the present study but for which further genomic investigations are needed.

The experimental design of the present study was largely inspired by the one in Naciff et al. (Naciff et al., 2005). Considering the lists of DEGs we identified, none were common to the lists they generated, at equivalent doses of treatment. Of note, they have used a previous version of the microarray used in the present study and comparison was only possible among Affymetrix probes associated to a gene ID, which represents only about 30% of the original lists (both studies identified many unknown transcripts). This difference between the two datasets is not surprising considering that the effects on germ cells in their study can be diluted in RNAs from predominantly somatic cells. Another major discrepancy is the route of exposure as in the present study pregnant rats were exposed by gavage compared to subcutaneous injections in theirs. EE2 and GE have short half-lifes after oral exposure in rats (Dusterberg et al., 1986; Kwon et al., 2007) and the actual levels of EE2 or GE that reached the fetus during gestation are unknown. Yet other developmental exposure studies using similar route of exposure at lower levels of EE2 (400 ng/kg/day) observed behavioural changes in offspring which strongly suggests that the chemical reached the fetus (Zaccaroni et al., 2016).

As perinatal germ cells express ERẞ (Rouiller-Fabre et al., 2015), they are potential direct targets of EE2 and GE. However, the effects on gene expression reported here are not major and the experimental design does not allow to elucidate whether they are due to direct effects in gonocytes, indirect effects via alteration of testicular somatic cells or untested paracrine or endocrine effects. On the other hand, it is interesting to note that our data suggest an increase in the number of altered genes between the two sampling time points, even though the treatment has ended. This suggests that treatment during gestation may have altered the regulatory mechanisms of gene expression in gonocytes, inducing changes in transcript levels in more differentiated germ cells at PND5. This could be due to genomic imprinting as suggested by others (Nilsson et al., 2022). Indeed, the window of exposure also corresponds to the time of epigenetic reprogramming in the mammalian male germline, when there is near complete erasure of DNA methylation (DNAme), followed by *de novo* DNAme. This process establishes germline-specific gene expression profiles but also, in the longer term, a key component of the sperm epigenome that will guide, in part, embryo development (Wu et al., 2015). Early exposures to EDCs have been shown to have long-term impact on the sperm epigenome, but the initial molecular mechanisms linking EDCs exposure to an abnormal germline epigenome are still unknown and would deserve to be studied in more details (Feil and Fraga, 2011; Pacchierotti and Spano, 2015; Wu et al., 2015).

## Data Availability

The datasets presented in this study can be found in online repositories. The names of the repository/repositories and accession number(s) can be found below: www.ebi.ac.uk/arrayexpress; under accession number E-MTAB-11591.

## Supplementary Material

Supplementary Figure S1 Analysis of DEGs overlap between groups. Venn Diagramms illustrating overlap after exposure to EE2 or GE at the two sampling stages, using **(A)** the total numbers of differentially expressed Olfactory receptor (Olr) coding genes, or **(B)** the total number of snoRNAs.

## References

[B1] Ahmad R. Gautam A. K. Verma Y. Sedha S. Kumar S. (2014). Effects of In Utero Di-butyl Phthalate and Butyl Benzyl Phthalate Exposure on Offspring Development and Male Reproduction of Rat. Environ. Sci. Pollut. Res. 21, 3156–3165. 10.1007/s11356-013-2281-x 24213843

[B2] Ali M. A. Wang Y. Qin Z. Yuan X. Zhang Y. Zeng C. (2021). Odorant and Taste Receptors in Sperm Chemotaxis and Cryopreservation: Roles and Implications in Sperm Capacitation, Motility and Fertility. Genes (Basel) 12. 10.3390/genes12040488 PMC8065900 33801624

[B3] Clermont Y. Perey B. (1957). The Stages of the Cycle of the Seminiferous Epithelium of the Rat: Practical Definitions in PA-Schiff-hematoxylin and Hematoxylin-Eosin Stained Sections. Rev. Can. Biol. 16, 451–462. 13528186

[B4] Clowney E. J. Magklara A. Colquitt B. M. Pathak N. Lane R. P. Lomvardas S. (2011). High-throughput Mapping of the Promoters of the Mouse Olfactory Receptor Genes Reveals a New Type of Mammalian Promoter and Provides Insight into Olfactory Receptor Gene Regulation. Genome Res. 21, 1249–1259. 10.1101/gr.120162.110 21705439 PMC3149492

[B5] Cronkhite J. T. Norlander C. Furth J. K. Levan G. Garbers D. L. Hammer R. E. (2005). Male and Female Germline Specific Expression of an EGFP Reporter Gene in a Unique Strain of Transgenic Rats. Dev. Biol. 284, 171–183. 10.1016/j.ydbio.2005.05.015 15993404

[B6] Delbès G. Levacher C. Duquenne C. Racine C. Pakarinen P. Habert R. (2005). Endogenous Estrogens Inhibit Mouse Fetal Leydig Cell Development via Estrogen Receptor Alpha. Endocrinology 146, 2454–2461. 10.1210/en.2004-1540 15661855

[B7] Delbès G. Levacher C. Habert R. (2006). Estrogen Effects on Fetal and Neonatal Testicular Development. Reproduction 132, 527–538. 10.1530/rep.1.01231 17008464

[B8] Delbes G. Blázquez M. Fernandino J. I. Grigorova P. Hales B. F. Metcalfe C. (2022). Effects of Endocrine Disrupting Chemicals on Gonad Development: Mechanistic Insights from Fish and Mammals. Environ. Res. 204, 112040. 10.1016/j.envres.2021.112040 34509487

[B9] Delbes G. Duquenne C. Szenker J. Taccoen J. Habert R. Levacher C. (2007). Developmental Changes in Testicular Sensitivity to Estrogens throughout Fetal and Neonatal Life. Toxicol. Sci. 99, 234–243. 10.1093/toxsci/kfm160 17569695

[B10] Delclos K. B. Weis C. C. Bucci T. J. Olson G. Mellick P. Sadovova N. (2009). Overlapping but Distinct Effects of Genistein and Ethinyl Estradiol (EE2) in Female Sprague-Dawley Rats in Multigenerational Reproductive and Chronic Toxicity Studies. Reprod. Toxicol. 27, 117–132. 10.1016/j.reprotox.2008.12.005 19159674 PMC2706590

[B11] Dupuis-Sandoval F. Poirier M. Scott M. S. (2015). The Emerging Landscape of Small Nucleolar RNAs in Cell Biology. WIREs RNA 6, 381–397. 10.1002/wrna.1284 25879954 PMC4696412

[B12] Düsterberg B. Kühne G. Täuber U. (1986). Half-lives in Plasma and Bioavailability of Ethinylestradiol in Laboratory Animals. Arzneimittelforschung 36, 1187–1190. 3778555

[B13] Feil R. Fraga M. F. (2011). Epigenetics and the Environment: Emerging Patterns and Implications. Nat. Rev. Genet. 13, 97–109. 10.1038/nrg3142 22215131

[B14] Gutendorf B. Westendorf J. (2001). Comparison of an Array of *In Vitro* Assays for the Assessment of the Estrogenic Potential of Natural and Synthetic Estrogens, Phytoestrogens and Xenoestrogens. Toxicology 166, 79–89. 10.1016/s0300-483x(01)00437-1 11518614

[B15] Habert R. Picon R. (1984). Testosterone, Dihydrotestosterone and Estradiol-17β Levels in Maternal and Fetal Plasma and in Fetal Testes in the Rat. J. Steroid Biochem. 21, 193–198. 10.1016/0022-4731(84)90383-2 6482429

[B16] Herrmann B. G. Koschorz B. Wertz K. Mclaughlin K. J. Kispert A. (1999). A Protein Kinase Encoded by the T Complex Responder Gene Causes Non-mendelian Inheritance. Nature 402, 141–146. 10.1038/45970 10647005

[B17] Huang da W. Sherman B. T. Lempicki R. A. (2009). Systematic and Integrative Analysis of Large Gene Lists Using DAVID Bioinformatics Resources. Nat. Protoc. 4, 44–57. 10.1038/nprot.2008.211 19131956

[B18] Iqbal K. Tran D. A. Li A. X. Warden C. Bai A. Y. Singh P. (2015). Deleterious Effects of Endocrine Disruptors Are Corrected in the Mammalian Germline by Epigenome Reprogramming. Genome Biol. 16, 59. 10.1186/s13059-015-0619-z 25853433 PMC4376074

[B19] Kang N. Koo J. (2012). Olfactory Receptors in Non-chemosensory Tissues. BMB Rep. 45, 612–622. 10.5483/bmbrep.2012.45.11.232 23186999 PMC4133803

[B20] Kuiper G. G. J. M. Carlsson B. Grandien K. Enmark E. Häggblad J. Nilsson S. (1997). Comparison of the Ligand Binding Specificity and Transcript Tissue Distribution of Estrogen Receptors α and β. Endocrinology 138, 863–870. 10.1210/endo.138.3.4979 9048584

[B21] Kwon S. H. Kang M. J. Huh J. S. Ha K. W. Lee J. R. Lee S. K. (2007). Comparison of Oral Bioavailability of Genistein and Genistin in Rats. Int. J. Pharm. 337, 148–154. 10.1016/j.ijpharm.2006.12.046 17280808

[B22] Law N. C. Oatley J. M. (2020). Developmental Underpinnings of Spermatogonial Stem Cell Establishment. Andrology 8, 852–861. 10.1111/andr.12810 32356598 PMC8324036

[B23] Lombó M. Herráez P. (2021). The Effects of Endocrine Disruptors on the Male Germline: an Intergenerational Health Risk. Biol. Rev. 96, 1243–1262. 10.1111/brv.12701 33660399

[B24] Manku G. Culty M. (2015). Mammalian Gonocyte and Spermatogonia Differentiation: Recent Advances and Remaining Challenges. Reproduction 149, R139–R157. 10.1530/rep-14-0431 25670871

[B25] Mann U. Shiff B. Patel P. (2020). Reasons for Worldwide Decline in Male Fertility. Curr. Opin. Urology 30, 296–301. 10.1097/mou.0000000000000745 32168194

[B26] Marlatt V. L. Bayen S. Castaneda-Cortès D. Delbès G. Grigorova P. Langlois V. S. (2022). Impacts of Endocrine Disrupting Chemicals on Reproduction in Wildlife and Humans. Environ. Res. 208, 112584. 10.1016/j.envres.2021.112584 34951986

[B27] Mulligan A. A. Welch A. A. Mctaggart A. A. Bhaniani A. Bingham S. A. (2007). Intakes and Sources of Soya Foods and Isoflavones in a UK Population Cohort Study (EPIC-Norfolk). Eur. J. Clin. Nutr. 61, 248–254. 10.1038/sj.ejcn.1602509 16943849

[B28] Naciff J. M. Hess K. A. Overmann G. J. Torontali S. M. Carr G. J. Tiesman J. P. (2005). Gene Expression Changes Induced in the Testis by Transplacental Exposure to High and Low Doses of 17α-Ethynyl Estradiol, Genistein, or Bisphenol A. Toxicol. Sci. 86, 396–416. 10.1093/toxsci/kfi198 15901920

[B29] Nilsson E. E. Ben Maamar M. Skinner M. K. (2022). Role of Epigenetic Transgenerational Inheritance in Generational Toxicology. Environ. Epigenet 8, dvac001. 10.1093/eep/dvac001 35186326 PMC8848501

[B30] O'Donnell L. Robertson K. M. Jones M. E. Simpson E. R. (2001). Estrogen and Spermatogenesis. Endocr. Rev. 22, 289–318. 10.1210/er.22.3.289 11399746

[B31] O'Shaughnessy P. J. Fowler P. A. (2011). Endocrinology of the Mammalian Fetal Testis. Reproduction 141, 37–46. 10.1530/rep-10-0365 20956578

[B32] Pacchierotti F. Spanò M. (2015). Environmental Impact on DNA Methylation in the Germline: State of the Art and Gaps of Knowledge. Biomed. Res. Int. 2015, 123484. 10.1155/2015/123484 26339587 PMC4538313

[B33] Picut C. A. Remick A. K. De Rijk E. P. C. T. Simons M. L. Stump D. G. Parker G. A. (2015). Postnatal Development of the Testis in the Rat. Toxicol. Pathol. 43, 326–342. 10.1177/0192623314547279 25217330

[B34] Reagan-Shaw S. Nihal M. Ahmad N. (2008). Dose Translation from Animal to Human Studies Revisited. FASEB J. 22, 659–661. 10.1096/fj.07-9574LSF 17942826

[B35] Reed C. E. Fenton S. E. (2013). Exposure to Diethylstilbestrol during Sensitive Life Stages: a Legacy of Heritable Health Effects. Birth Defect Res. C 99, 134–146. 10.1002/bdrc.21035 PMC3817964 23897597

[B36] Robaire B. Delbes G. Head J. A. Marlatt V. L. Martyniuk C. J. Reynaud S. (2022). A Cross-Species Comparative Approach to Assessing Multi- and Transgenerational Effects of Endocrine Disrupting Chemicals. Environ. Res. 204, 112063. 10.1016/j.envres.2021.112063 34562476

[B37] Rouiller-Fabre V. Guerquin M. J. Nâ€™Tumba-Byn T. Muczynski V. Moison D. Tourpin S. (2015). Nuclear Receptors and Endocrine Disruptors in Fetal and Neonatal Testes: a Gapped Landscape. Front. Endocrinol. 6, 58. 10.3389/fendo.2015.00058 PMC4423451 25999913

[B38] Rwigemera A. El Omri-Charai R. Lecante L. L. Delbes G. (2021). Dynamics in the Expression of Epigenetic Modifiers and Histone Modifications in Perinatal Rat Germ Cells during De Novo DNA Methylation†. Biol. Reprod. 104, 361–373. 10.1093/biolre/ioaa206 33324985

[B39] Rwigemera A. Joao F. Delbes G. (2017). Fetal Testis Organ Culture Reproduces the Dynamics of Epigenetic Reprogramming in Rat Gonocytes. Epigenetics Chromatin 10, 19. 10.1186/s13072-017-0127-3 28413450 PMC5387332

[B40] Saitou M. Yamaji M. (2012). Primordial Germ Cells in Mice. Cold Spring Harb. Perspect. Biol. 4, 8375. 10.1101/cshperspect.a008375 PMC3536339 23125014

[B41] Schwartz C. L. Christiansen S. Vinggaard A. M. Axelstad M. Hass U. Svingen T. (2019). Anogenital Distance as a Toxicological or Clinical Marker for Fetal Androgen Action and Risk for Reproductive Disorders. Arch. Toxicol. 93, 253–272. 10.1007/s00204-018-2350-5 30430187

[B42] Skakkebaek N. E. Rajpert-De Meyts E. Buck Louis G. M. Toppari J. Andersson A.-M. Eisenberg M. L. (2016). Male Reproductive Disorders and Fertility Trends: Influences of Environment and Genetic Susceptibility. Physiol. Rev. 96, 55–97. 10.1152/physrev.00017.2015 26582516 PMC4698396

[B43] Skinner M. K. (2016). Epigenetic Transgenerational Inheritance. Nat. Rev. Endocrinol. 12, 68–70. 10.1038/nrendo.2015.206 26585656 PMC5287152

[B44] Skinner M. K. Haque C. G.-B. M. Nilsson E. Bhandari R. McCarrey J. R. Mccarrey J. R. (2013). Environmentally Induced Transgenerational Epigenetic Reprogramming of Primordial Germ Cells and the Subsequent Germ Line. PLoS One 8, e66318. 10.1371/journal.pone.0066318 23869203 PMC3712023

[B45] van den Heuvel M. W. Van Bragt A. J. M. Alnabawy A. K. M. Kaptein M. C. J. (2005). Comparison of Ethinylestradiol Pharmacokinetics in Three Hormonal Contraceptive Formulations: the Vaginal Ring, the Transdermal Patch and an Oral Contraceptive. Contraception 72, 168–174. 10.1016/j.contraception.2005.03.005 16102549

[B46] Walker C. Garza S. Papadopoulos V. Culty M. (2021). Impact of Endocrine-Disrupting Chemicals on Steroidogenesis and Consequences on Testicular Function. Mol. Cell. Endocrinol. 527, 111215. 10.1016/j.mce.2021.111215 33657436

[B47] Walker C. Ghazisaeidi S. Collet B. Boisvert A. Culty M. (2020). In Utero exposure to Low Doses of Genistein and Di‐(2‐ethylhexyl) Phthalate (DEHP) Alters Innate Immune Cells in Neonatal and Adult Rat Testes. Andrology 8, 943–964. 10.1111/andr.12840 32533902

[B48] Wallen W. J. Belanger M. P. Wittnich C. (2001). Sex Hormones and the Selective Estrogen Receptor Modulator Tamoxifen Modulate Weekly Body Weights and Food Intakes in Adolescent and Adult Rats. J. Nutr. 131, 2351–2357. 10.1093/jn/131.9.2351 11533278

[B50] Welsh M. Suzuki H. Yamada G. (2014). The Masculinization Programming Window. Endocr. Dev. 27, 17–27. 10.1159/000363609 25247641

[B51] Wilhelm D. Palmer S. Koopman P. (2007). Sex Determination and Gonadal Development in Mammals. Physiol. Rev. 87, 1–28. 10.1152/physrev.00009.2006 17237341

[B52] Wu H. Hauser R. Krawetz S. A. Pilsner J. R. (2015). Environmental Susceptibility of the Sperm Epigenome during Windows of Male Germ Cell Development. Curr. Envir Health Rpt 2, 356–366. 10.1007/s40572-015-0067-7 PMC4623071 26362467

[B53] Zaccaroni M. Seta D. D. Farabollini F. Fusani L. Dessì-Fulgheri F. (2016). Developmental Exposure to Very Low Levels of Ethynilestradiol Affects Anxiety in a Novelty Place Preference Test of Juvenile Rats. Neurotox. Res. 30, 553–562. 10.1007/s12640-016-9645-1 27358038

