# Supplementary figures and images for: Impact of in Utero Rat Exposure to 17Alpha-Ethinylestradiol or Genistein on Testicular Development and Germ Cell Gene Expression
Source: Front Toxicol. 2022 Jun 2;4:893050. doi: 10.3389/ftox.2022.893050 (PMC9201280; doi:10.3389/ftox.2022.893050)

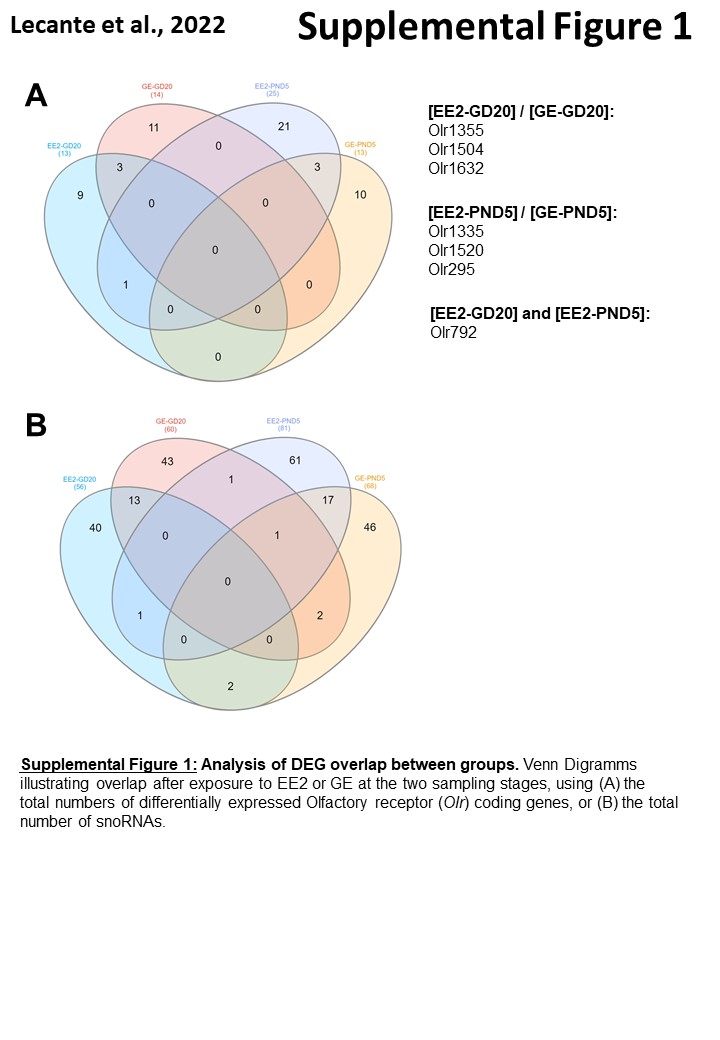

Supplement: Supplementary file 1 [file Image1.JPEG]
