# Supplementary material for: Impact of in Utero Rat Exposure to 17Alpha-Ethinylestradiol or Genistein on Testicular Development and Germ Cell Gene Expression
Source: Front Toxicol. 2022 Jun 2;4:893050. doi: 10.3389/ftox.2022.893050 (PMC9201280; doi:10.3389/ftox.2022.893050)
Supplement: Supplementary file 2 [file Table1.DOC]

**Supplemental Table 1: Cell viability and purity before and after cell sorting.**

|  | | **Before sorting** | **GFP-positive cells fraction** | |
| --- | --- | --- | --- | --- |
|  |  | Viability (%) | Purity (%) | Viability (%) |
| **GD20** | CTRL (n=5) | 97.91 ± 0.39 | 86.11 ± 5.58 | 86.24 ± 5.60 |
|  | EE2 (n=4) | 97.14 ± 1.20 | 91.48 ± 1.64 | 91.51 ± 2.19 |
|  | GE (n=4) | 96.34 ± 0.32 | 88.15 ± 1.75 | 89.29 ± 2.65 |
| **PND5** | CTRL (n=4) | 95.76 ± 0.77 | 94.52 ± 3.05 | 94.95 ± 2.93 |
|  | EE2 (n=4) | 96.27 ± 0.91 | 94.83 ± 2.19 | 95.68 ± 1.80 |
|  | GE (n=4) | 94.91 ± 2.56 | 91.51 ± 4.05 | 95.00 ± 1.33 |
